# 3plex Web: An interactive platform for RNA:DNA triplex prediction and analysis

**DOI:** 10.1016/j.csbj.2025.07.005

**Published:** 2025-07-14

**Authors:** Marco Masera, Chiara Cicconetti, Francesca Ferrero, Salvatore Oliviero, Ivan Molineris

**Affiliations:** Dipartimento di Scienze della Vita e Biologia dei Sistemi and MBC, Università di Torino, Via Accademia Albertina 13, Torino, 10126, TO, Italy

**Keywords:** DNA, RNA, RNA–DNA interaction, Triplex, Long non-coding RNA, lncRNA, Gene regulation, Web application

## Abstract

Long non-coding RNAs (lncRNAs) exert their functions by cooperating with other molecules, including proteins and DNA. Triplexes, formed through the interaction between a single-stranded RNA (ssRNA) and a double-stranded DNA (dsDNA), have been consistently described as a mechanism that allows lncRNAs to target specific genomic sequences in vivo. Building on the computational tool 3plex, we developed 3plex Web, an accessible platform that enhances the prediction of RNA:DNA triplexes by integrating interactive visualization, statistical evaluation, and user-friendly downstream analysis workflows. 3plex Web implements new features such as input randomization for statistical assessments, interactive profile plotting for triplex stability, and customizable DNA Binding Domain (DBD) selection. This platform enables rapid analysis through PATO, substantially reducing processing times compared to previous methods, while offering Snakemake workflows to integrate gene expression data and explore lncRNA regulatory mechanisms.

## Graphical abstract

**Figure:**
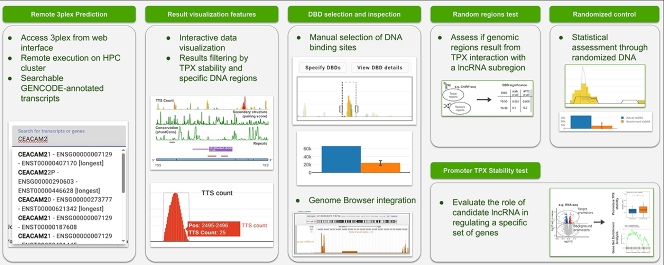

## 1 Introduction

Triplexes are molecular complexes resulting from the interaction of ssRNA and dsDNA molecules. The formation of these structures requires the Watson-Crick interacting nucleotides of DNA to establish an additional hydrogen bond with the nucleotides of the RNA (Hoogsteen pairing) [13]. Triplexes principally occur following a canonical set of rules: a purine-rich portion of the DNA can bind to a pyrimidinic or mixed portion of the RNA in a parallel direction [C:G-C, U:A-T, G:G-C] and, alternatively, a purinic or mixed portion of the RNA in an anti-parallel direction [G:G-C, A:A-T, U:A-T] [8]. The triplex-forming subsequence on the ssRNA is denoted as Triplex Forming Oligonucleotide (TFO), and its cognate substring on the dsDNA is named Triplex Target Site (TTS). Taken together, the TSS and the associated TFO combine to form a triplex (TPX).

TPX formation has been consistently described as a mechanism that allows lncRNAs to target specific genomic sequences *in vivo* and regulate gene expression [16], [21], [12]. We previously developed 3plex [2] which enhanced the state-of-the-art TPX prediction algorithm (Triplexator [1]) by incorporating relevant biophysical properties of these structures. In fact, 3plex differentiates itself from other methods by enriching sequence-based prediction with data from denaturation experiments, which is fundamental to determine the thermal stability of structures [6], [23]. The importance of this measurement is demonstrated by recent research that has expanded its understanding [3]. Furthermore, 3plex incorporates prediction of secondary RNA structure [15].

In this paper, we present 3plex Web, a refined and enhanced version of 3plex, designed to elevate the precision, efficiency, and accessibility of RNA:DNA:DNA TPX prediction and analysis.

In 3plex Web, we enable the remote execution of analyses with a web interface, without the need for complex software installations or advanced computational skills, thereby making TPX analysis accessible to all biologists, while offering a set of interactive visualization tools for navigating and interpreting the results. 3plex Web also introduces a randomization procedure for statistical evaluation of the biological significance of predicted TPXs.

Moreover, we have developed a suite of predefined Snakemake workflows that easily integrate 3plex results with omics data, such as RNA-seq and ChIRP-seq, to explore the regulatory potential of lncRNAs. In addition to the raw TPX predictions from the 3plex analysis, these workflows can highlight the importance of TPX formation in specific contexts and identify the most relevant DNA-binding domains. The Snakemake workflows are accessible via the command-line interface, with selected features integrated into the web platform for enhanced usability. Lastly, 3plex achieves a 100-fold increase in speed by replacing Triplexator with PATO [14], a reimplementation of Triplexator that significantly reduces runtime, enabling the use of computationally expensive yet more accurate prediction parameters we already elucidated in our previous work [2].

Our previous work demonstrated that 3plex outperforms existing tools, including Triplexator and TriplexAligner [22], regarding prediction accuracy. Compared to 3plex Web, TRIPBASE [14] provides a precomputed database of TPX predictions throughout the human genome. It offers remote access to the Triplexator predictor, which is slower and less accurate than 3plex, shows accessibility problems, and has limited interactive exploration capabilities. Triplex Domain Finder [9] requires complex local installation of software and reference genomic data; it is based on Triplexator, does not include thermal stability or secondary structure considerations, and has limited interactive exploration capabilities. Genna et al. recently developed a new TPX predictor based on machine learning approaches [3], but their software and model parameters are not yet published for comparisons or applications in different contexts.

## 2 Methodology

### 2.1 3plex Web

3plex Web provides access to the 3plex core via a web interface, allowing for remote TPX prediction and offering additional statistical and data navigation features. The application is composed of four components: i) a web client built with the Angular framework, ii) a public API server (frontend) based on Django REST, iii) a private backend server, and iv) the core 3plex workflow. The web app offers a graphic interface and functions as the user's entry point for the application. The frontend server provides public APIs to the client, manages user data, supports data visualization, caches results, and securely stores job information. The backend is a stateless server deployed on an HPC cluster, responsible for queuing 3plex jobs via SLURM and delivering results upon completion. The jobs are then executed by the 3plex core workflow, enclosed in a Singularity container, on the first available node.

The 3plex core workflow, implemented in Snakemake [10] and leveraging Snakemaker [17], has been optimized by integrating PATO [4], improving computational efficiency compared to the previous Triplexator-based implementation. PATO maintains the canonical set of rules for TPX identification while significantly reducing computation time and delivering easily interpretable output. A new randomization feature predicts TPXs on a large set of random permutations of the original dsDNA sequence, functioning as a control against the original one.

**Fig. 1:**
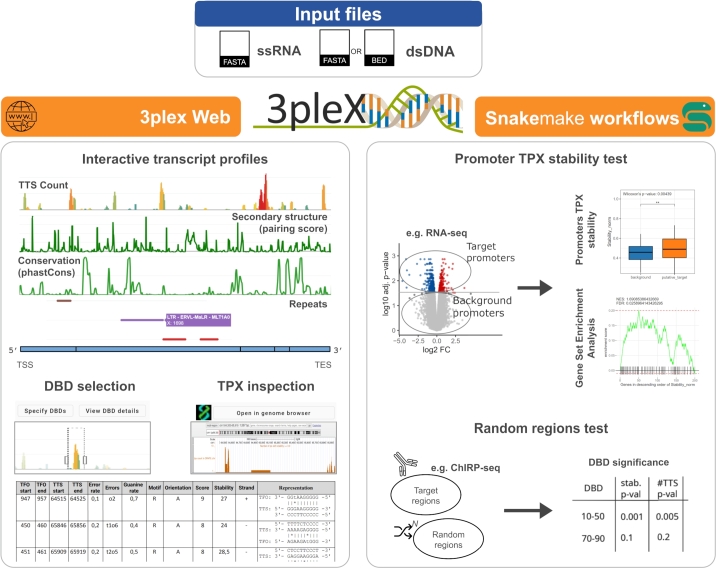
Starting from an ssRNA sequence and a set of dsDNA sequences (or regions), 3plex Web predicts all the TPX interactions and dynamically visualizes the results. The profiles display key features along the ssRNA, including the TTS count, nucleotide pairing score (secondary structure), conservation, and repetitive elements annotations. These plots are filterable according to the thermal stability of the TPXs, and DBDs can be dynamically defined and inspected. 3plex Web results can be directly integrated into the Genome Browser. Snakemake workflows facilitate the statistical evaluation of the identified TPXs. The promoter TPX stability test compares the stability of the TPXs formed with promoters of genes of interest with all the remaining genes (Wilcoxon's test) and performs a gene set enrichment analysis. The random region test compares the stability of the DBDs' putative triplexes identified considering a set of target regions (e.g., ChIRP-seq data) with a null distribution built on the stability scores obtained by testing randomized genomic regions N times.

To investigate the propensity of ssRNA regions to form biologically relevant TPXs, 3plex Web provides useful visualization functionalities (Fig. 1). The “TTS count” plot displays the total number of TTSs identified along the ssRNA sequence together with the thermal stability of the best predicted TPX. If the randomization procedure is enabled, the plot also displays the expected TTS count in randomized sequences and reports empirical p-values. Although highly stable TPXs typically involve long stretches of the ssRNA, we previously demonstrated that short and less stable TPXs can play regulatory roles in gene expression, like transcription factor binding events [16], [2], [5]. To accommodate these considerations, 3plex Web allows users to dynamically adjust the stability threshold in real-time, facilitating an exploratory analysis of TPX formation potential across the entire ssRNA sequence.

Since TFOs are expected to be formed by unfolded portions of the ssRNA, the “Secondary structure” plot displays the propensity of the sequence to be double-stranded in the RNA secondary structure [15]. If human or mouse species is selected, two additional plots visualize the sequence phylogenetic conservation score [19] and the repetitive element position.

To assist in the identification of functional TPX-forming regions, 3plex Web enables the interactive detection of DNA Binding Domains (DBDs)—ssRNA regions predicted to engage in sequence-specific TPXs. In contrast with other tools like Triplex Domain Finder [9], which automatically define the DBDs, 3plex Web provides users with the flexibility to manually adjust DBD boundaries, refining their position and width for more tailored analyses. For each DBD, 3plex Web retrieves the complete list of TFO-TTS interactions in tabular format, available for download or online analysis. For human and mouse species, 3plex offers a searchable table detailing key properties for each dsDNA region, including genomic coordinates, associated genes, best stability score, best PATO score, and normalized thermal stability. Upon selection of a specific dsDNA region, 3plex Web dynamically updates the profile plots, restricting the analysis to TPXs associated with the chosen genomic region. Additionally, a direct link to the UCSC Genome Browser allows users to inspect the predicted TPX binding sites within their genomic context. A full overview of the 3plex Web interface is provided in Supplementary Material S1.

Interactive data visualization is supported by a set of indexes and data structured produced by the updated version of the 3plex Core; in particular an additional data structure summarizes TTS counts across all stability thresholds for each segment of the ssRNA sequence; these TPX profiles are stored as sparse matrices and compressed in msgpack format, enabling direct access via the web client. A second sparse matrix encodes the averages, variances and quartiles of expected TPX counts over the randomized DNAs.

### 2.2 3plex Snakemake workflows

Investigating the mechanism of action of lncRNAs requires assessing the biological significance of their predicted interactions with target molecules to formulate meaningful hypotheses [9]. To support this, we have implemented downstream analysis pipelines that provide insights into the TPX-mediated functionality of an ssRNA. These pipelines enable the integration of omics data, such as RNA-seq and ChIRP-seq, which are commonly employed to explore lncRNA function.

The random region test determines whether a specific set of genomic regions (e.g., ChIRP-seq peaks) could arise from a TPX-mediated interaction with a specific lncRNA subregion, i.e., a DBD. To assess this, N randomized versions of the target regions are generated using bedtools shuffle [20], excluding ENCODE blacklists and genomic gaps. 3plex is then executed on both the original target regions and the randomized regions, and the TTS count and thermal stability are computed for each nucleotide of the ssRNA. DBDs are identified based on the overlapping TFOs found in the target regions. For each DBD, the upper quartile of the TTS count and the thermal stability of the included nucleotides are extracted. These values are then compared with those derived from the randomized control regions to compute empirical p-values, providing statistical significance for TPX-mediated interactions.

The promoter TPX stability test evaluates the potential role of a candidate ssRNA in regulating a specific set of genes, such as those identified as differentially expressed in knock-out experiments. By integrating gene expression data, this workflow assesses whether TPXs are enriched at regulated gene promoters. Users provide a background gene set (e.g., all the expressed genes in the system), a list of genes of interest (e.g., differentially expressed genes following ssRNA knockout), and the ssRNA sequence. Promoters are pre-defined as spanning -1500 to +500 bp relative to transcription start sites, based on Matched Annotation from NCBI and EMBL-EBI (MANE) [18] 3plex predicts TPX formation with these promoters and evaluates the stability of interactions between the ssRNA and promoters of genes of interest compared to all other genes using a Mann-Whitney test. Additionally, a Gene Set Enrichment Analysis [7], [11] ranks genes by their TPX stability score, assessing the enrichment significance of high or low stability interactions at the promoters of selected genes. The leading-edge table provides a selection of candidate target genes for further investigation.

Both pipelines are designed for command-line execution using Snakemake. Additionally, the promoter TPX stability test is integrated into 3plex Web for remote job execution.

## 3 Overview

3plex Web allows access to the 3plex prediction feature via a web interface. Users can navigate to the website and submit new jobs to the 3plex core, specifying: 1) one ssRNA sequence in FASTA format and 2) a set of dsDNA sequences in multi-FASTA or BED format. For the human and mouse species, the ssRNA input can be selected from the searchable list of GENCODE-annotated transcripts, while the dsDNA sequences can be set to a list of promoters based on Matched Annotation from NCBI and EMBL-EBI (MANE) [18]. Users can adjust key parameters influencing TPX prediction, such as the minimum TPX length and masking of the ssRNA based on the secondary structure prediction. Optionally, the randomization procedure can be enabled, generating a randomized set of dsDNA sequences, enabling a robust statistical comparison between observed TTS profiles and expected distributions.

Upon job submission, the platform generates a unique token, allowing users to retrieve their results when available. Alternatively, users can provide an email address to receive a notification once the job is complete.

The job overview page allows downloading the raw output data of the 3plex core and the Snakemake logs produced during execution, while the data visualization page offers interactive visualization features.

In the data visualization page, the four plots with TPX profile, secondary structure, conservation and repetitive elements are shown to the user. These plots can be dynamically updated with the Minimum Stability Threshold slider displayed at the top of the page.

The user can select DBDs using a dedicated button and simply dragging on the profile plot with the mouse to select an area of the sequence. The View DBD Details button enables the selection of one DBD and visualization of its full TPX table, as well as an empirical p-value computed against randomized sequences.

Finally, if the dsDNA sequences were provided in BED format or using a set of pre-defined promoters, a searchable table at the bottom of the screen displays the various dsDNA sequences with the best stability, the normalized stability, and the best scores between TPXs produced in this subsection. Entries in the table can be clicked, opening a new data visualization page with results restricted to TPXs produced on the subsequence. This page offers the same functionalities of the main data visualization page, plus the visualization and export of the full TPX table and the possibility to open the TPX profile on the dsDNA sequence on the Genome Browser. Job data is retained on the server for up to four weeks; users can export backups of the job data and use them to restore expired jobs.

Command-line functionalities leverage the Snakemake workflow manager and are distributed via GitHub. For a seamless environment setup, a Singularity definition file is provided together with shell scripts to build and run the container. If Singularity is installed, the environment can be set up with a single shell command, using the provided script. Alternatively, the user can set up 3plex manually following the guidelines provided in the repository README. Once the environment is set up, the workflows can be executed by running the Snakemake rules *run_promoter_tpx_stability_test* and *run_random_region_test*. A test directory is provided with a main template for testing the 3plex features.

## 4 Conclusions

3plex Web enables access to the 3plex core and remote 3plex execution via a web interface, allowing users to run triplex prediction without the need for computational resources or skills, reducing the barrier to access TPX analysis. While raw 3plex results can be downloaded, the data visualization features allow for flexible exploration of the results, the definition of custom DNA binding domains (DBDs), and the integration with the Genome Browser. The randomization feature integrates statistical significance into the model's predictions.

3plex Snakemake workflows facilitate the assessment of the functional activity of predicted TPXs by integrating experimental data. This approach is particularly beneficial when exploring the regulatory roles of lncRNAs, offering a list of the most relevant DBDs and the significance of lncRNA activity on selected genomic regions. These workflows can be easily executed with both Singularity and Snakemake, providing a valuable resource to explore the molecular functions of candidate RNAs.

## Funding

This project has been made possible in part by grant 2024-342822 from the Chan Zuckerberg Initiative DAF, an advised fund of 10.13039/100000923Silicon Valley Community Foundation.

## CRediT authorship contribution statement

**Marco Masera:** Writing – original draft, Software, Methodology, Conceptualization. **Chiara Cicconetti:** Writing – original draft, Software, Methodology, Conceptualization. **Francesca Ferrero:** Methodology. **Salvatore Oliviero:** Supervision, Project administration. **Ivan Molineris:** Writing – review & editing, Supervision, Project administration, Funding acquisition.

## Declaration of Competing Interest

None declared.

## Data Availability

3plex Web is freely available at https://3plex.unito.it as an on-line web service. The source code for 3plex is available at https://github.com/molinerisLab/3plex, and it is paired with a definition file to setup the application in a Singularity image.

## Appendix A Supplementary material

MMC Overview of 3plex Web interface. MMC
