# Supplementary material for: 3plex Web: An interactive platform for RNA:DNA triplex prediction and analysis
Source: Comput Struct Biotechnol J. 2025 Jul 14;27:3110–3. doi: 10.1016/j.csbj.2025.07.005 (PMC12284569; doi:10.1016/j.csbj.2025.07.005)
Supplement: MMC — Overview of 3plex Web interface. [file mmc1.pdf]

3plex Web - Web Interface Overview

Data visualization, main components

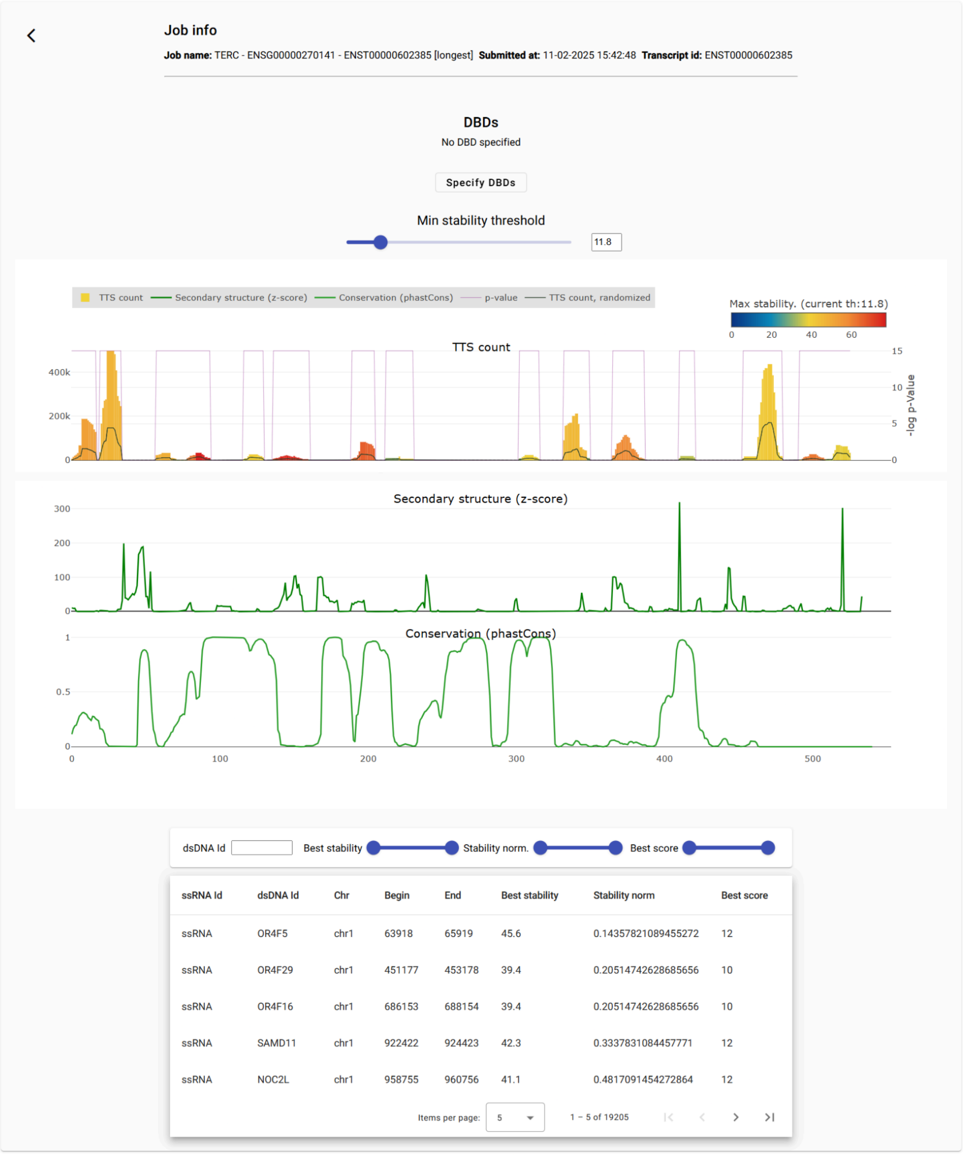

Example of 3plex Web data visualization page. ssRNA: TERC - ENSG00000270141 - ENST00000602385; dsDNA: MANE; Randomization: 10 iterations; Default 3plex parameters).

The TTS Count plot illustrates the number of TPXs formed at each position along the ssRNA sequence. The color gradient represents the maximum thermal stability of TPXs at each position. The green line, superimposed on the plot, indicates the median value of the same measure, calculated from randomized dsDNA tracks.
The following plots show the secondary structure, and the table at the bottom displays a row for each sequence in the dsDNA BED file. Users can select a sequence to view the corresponding results, showing only TPXs formed on that sequence. Additionally, they can visualize the TTS count within the genome browser.

DBD selection

DBDs

327 - 350

Select a DBD to view the details

Exit DBD view

Min stability threshold

12.4

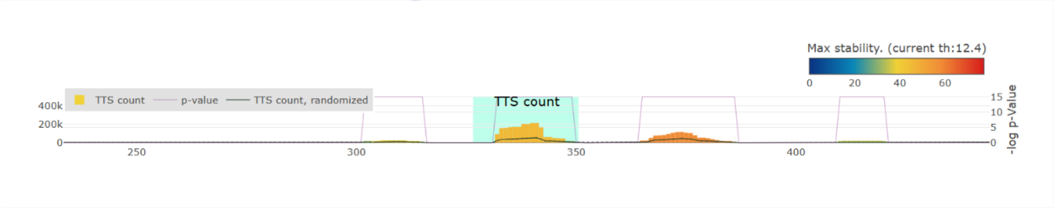

Selected DBD: 327-350

CTCTCGGGGGCGAGGGCGAGGTT

TTS count - actual dsDNA compared to randomized tracks

p-value: 0.00006699899783280117

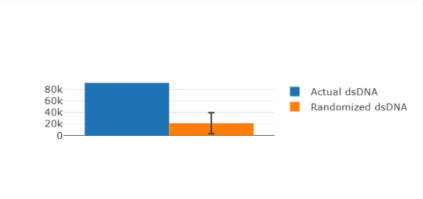

All TPX table

Export in excel

<table>
<tr><th>Duplex ID</th><th>TFO start</th><th>TFO end</th><th>TTS start</th><th>TTS end</th><th>Error rate</th><th>Errors</th><th>Guanine rate</th><th>Motif</th><th>Orientation</th><th>Score</th><th>Stability</th><th>Strand</th><th>Representation</th></tr>
<tr><td>ACOT7 - chr1</td><td>332</td><td>342</td><td>6393572</td><td>6393582</td><td>0.2</td><td>d2b4</td><td>0.8</td><td>R</td><td>A</td><td>8</td><td>24</td><td>+</td><td>TFO: 3'- GGAGcG
||*|*|
TTS: 5'- GGcGtG
3'- CCGCAC</td></tr>
<tr><td>ACOT7 - chr1</td><td>333</td><td>343</td><td>6393865</td><td>6393875</td><td>0.2</td><td>d2b5</td><td>0.7</td><td>R</td><td>A</td><td>8</td><td>24</td><td>+</td><td>TFO: 3'- GGGAGc
||*||*
TTS: 5'- GGcAGt
3'- CCGTCA</td></tr>
<tr><td>ACOT7 - chr1</td><td>333</td><td>343</td><td>6393486</td><td>6393496</td><td>0.2</td><td>b4t6</td><td>0.8</td><td>R</td><td>A</td><td>8</td><td>26</td><td>-</td><td>5'- CCCCGC
TTS: 3'- GGGGcG
||||*|
TFO: 5'- GGGGcG</td></tr>
<tr><td>ACOT7 - chr1</td><td>332</td><td>342</td><td>6393572</td><td>6393582</td><td>0.2</td><td>d2b4</td><td>0.8</td><td>R</td><td>A</td><td>8</td><td>24</td><td>+</td><td>5'- [illegible]
||*|*|
TTS: 5'- GGcGtG
3'- CCGCAC</td></tr>
<tr><td>ACOT7 - chr1</td><td>333</td><td>343</td><td>6393865</td><td>6393875</td><td>0.2</td><td>d2b5</td><td>0.7</td><td>R</td><td>A</td><td>8</td><td>24</td><td>+</td><td>TFO: 3'- GGGAGc
||*||*
TTS: 5'- GGcAGt
3'- CCGTCA</td></tr>
<tr><td>ACOT7 - chr1</td><td>333</td><td>343</td><td>6393486</td><td>6393496</td><td>0.2</td><td>b4t6</td><td>0.8</td><td>R</td><td>A</td><td>8</td><td>26</td><td>-</td><td>5'- CCCCGC
TTS: 3'- GGGGcG
||||*|
TFO: 5'- GGGGcG</td></tr>
<tr><td>ACOT7 - chr1</td><td>332</td><td>342</td><td>6393491</td><td>6393501</td><td>0.2</td><td>b5d7</td><td>0.8</td><td>R</td><td>A</td><td>8</td><td>25</td><td>-</td><td>5'- CCCCCG
TTS: 3'- GGGGGc
|||||*
TFO: 5'- GGGGGc</td></tr>
<tr><td>ACOT7 - chr1</td><td>338</td><td>348</td><td>6393737</td><td>6393747</td><td>0.2</td><td>d3o5</td><td>0.6</td><td>R</td><td>A</td><td>8</td><td>26</td><td>-</td><td>5'- CTCACT
TTS: 3'- GAGtGA
|||*|*
TFO: 5'- GAGGGc</td></tr>
</table>

Items per page: 5

1 – 5 of 211670

The user manually specified a DBD region on the ssRNA profile track. In the DBD viewer, a plot displays the TPX count on the specified dsDNA track compared to the average TPX count on randomized dsDNA tracks. A p-value is computed on this observed difference. A table shows all TPXs in the selected region, exportable in *csv* format.

Genome browser integration

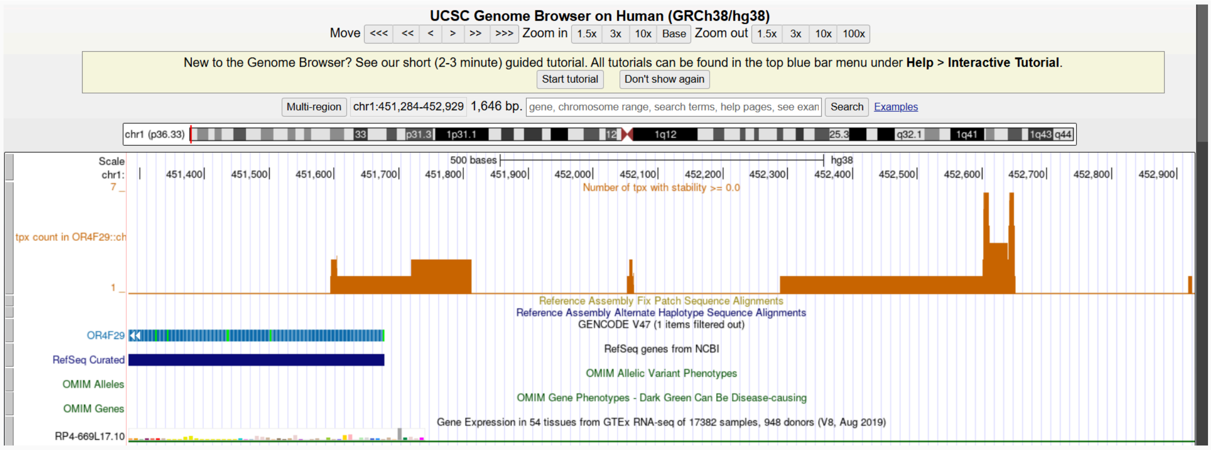

Example of genome browser integration. A specific dsDNA sequence has been selected and opened on the Genome Browser. A TTS count plot is computed on the dsDNA track.
